# A molecular epidemiological study on *Escherichia coli* in young chicks with colibacillosis identified two possible outbreaks across farms

**DOI:** 10.1186/s13567-023-01140-6

**Published:** 2023-02-06

**Authors:** Inger Helene Kravik, Håkon Kaspersen, Siri Kulberg Sjurseth, Katharine Rose Dean, Bruce David, Marina Aspholm, Camilla Sekse

**Affiliations:** 1 Norwegian Veterinary Institute, Ås, Norway; 2 Nortura SA, Økern, P.O. Box 360, 0513 Oslo, Norway; 3 Faculty of Veterinary Medicine, The Norwegian University of Life Sciences, Postboks 5003, 1432 Ås, Norway

**Keywords:** Avian pathogenic *Escherichia coli* (APEC), colibacillosis, poultry, whole genome sequencing, virulence-associated genes (VAGs), outbreak, systematic sampling

## Abstract

Avian pathogenic *Escherichia coli* (APEC) is the cause of colibacillosis outbreaks in young poultry chicks, resulting in acute to peracute death. The high morbidity and mortality caused by colibacillosis results in poor animal welfare, reduced sustainability and economical loss worldwide. To advance the understanding of the molecular epidemiology, genomic relatedness and virulence traits of APEC, we performed systematic sampling from 45 confirmed colibacillosis broiler flocks with high first week mortality (FWM) during 2018–2021. From these flocks, 219 APEC isolates were whole genome sequenced (WGS) and bioinformatic analyses were performed. The bioinformatic analyses included sequence typing (ST), serotyping, detection of virulence-associated genes (VAGs) and phylogenetic analysis. Our results showed a high prevalence of ST23, ST429 and ST95 among APEC isolates from Norwegian broiler flocks, and identified ST23, ST429, ST117 and ST371 to cause disease more often alone, compared to ST95, ST69 and ST10. Phylogenetic analyses, together with associated metadata, identified two distinct outbreaks of colibacillosis across farms caused by ST429 and ST23 and gave insight into expected SNP distances within and between flocks identified with the same ST. Further, our results highlighted the need for combining two typing methods, such as serotyping and sequence typing, to better discriminate strains of APEC. Ultimately, systematic sampling of APEC from multiple birds in a flock, together with WGS as a diagnostic tool is important to identify the disease-causing APEC within a flock and to detect outbreaks of colibacillosis across farms.

**Supplementary Information:**

The online version contains supplementary material available at 10.1186/s13567-023-01140-6.

## Introduction

Colibacillosis is considered a disease of high importance in poultry production as it gives rise to poor animal welfare and high economic losses worldwide. It is caused by avian pathogenic *Escherichia coli* (APEC) which is classified as an extra-intestinal pathogenic *E. coli* (ExPEC), together with neonatal meningitis-causing *E. coli* (NMEC) and uropathogenic *E. coli* (UPEC). Colibacillosis is suspected when the mortality among young chicks in a flock increases rapidly during the first week after hatching, usually peaking between two to 5 days after hatching. At necropsy, the chicks show typical macroscopic lesions associated with acute to per acute polyserositis, such as an enlarged spleen, edematous serous membranes and umbilicus. At a later stage of the disease, colisepticemia may lead to fibrinous polyserositis with fibrin exudates covering the serosa of the inner organs like the liver (perihepatitis) and heart (pericarditis). Isolation and identification of *E. coli* confirms the diagnosis [1].

Extra-intestinal pathogenic *E. coli*, including APEC, is considered a diverse group of pathogens, and several studies have attempted to group and identify APEC according to virulence-associated properties [2–4]. Historically, *E. coli* has been serogrouped by detecting somatic O–antigens with antisera. The most common APEC serogroups are O1, O2, and O78 [1, 5]. Later on, phylogrouping, a triplex PCR method to group *E. coli* based on the presence or absence of three genes divides *E. coli* into seven phylogroups: A, B1, B2, C, D, E and F, have been used [6]. MLST is another common method for typing *E. coli,* including APEC, and it is based on a combination of seven housekeeping genes in the *E. coli* genome [7]. Some of the most common APEC STs identified in Europe include ST23, ST69, ST95, ST117, ST131, ST140 and ST428/429 [5].

Whole genome sequencing (WGS), on the other hand, enables an array of in silico characterization methods, such as MLST, serotyping, characterization of VAGs and phylogenetic analysis [5, 8]. Core gene analysis and phylogenetic analysis, detect variations at nucleotide level and may be used to study relatedness, the latter being useful to detect and confirm possible outbreaks and their origin. Phylogenetic methods, however, are continuously developing and the outcomes of such molecular analyses require in-depth genomic understanding, essential bioinformatic knowledge as well as understanding of the pathogens to be studied [9, 10].

Between 2014 and 2016 the Nordic countries, including Norway, experienced a sudden increase in flocks diagnosed with colibacillosis [11]. Whole genome sequencing and genomic investigation of isolates from the affected flocks revealed a predominant lineage of ST117 O78:H4, but also a genetically diverse population. The experience highlighted the need for more knowledge of the molecular epidemiology of APEC in the Norwegian broiler production. In 2018, a systematic sampling of broiler flocks with high first week mortality (FWM) was therefore initiated.

The aim of this study was to systematically collect APEC isolates from Norwegian broiler flocks with high FWM and use WGS and bioinformatic analysis for in depth characterization and comparison of isolates from the same flock and between flocks. Flock-related metadata such as sampling date, hatchery, parent-flocks and hybrid were collected at each sampling occasion, enabling detection of potential association between APEC types identified. Finally, the relationship between the identified STs and their serotype- and VAG- profiles were studied.

## Materials and methods

### Study design: Necropsy and sampling

Flocks with predicted FWM above 2% elicited sampling from a poultry veterinarian. The flocks were from different regions of Norway and of different hybrids, though the predominant hybrid in Norway is at present Ross 308 (Table 1). Necropsies on ten birds/flock, recently died or euthanized for animal welfare reasons, were performed by a poultry veterinarian. Macroscopic lesions were noted as present/absent in a predefined submission form. Five of the ten examined birds with the most typical macroscopic lesions associated with colibacillosis were selected for bacteriological examination from the spleen, liver and one other organ as previously described in Kravik et al. [12].

**Table 1:** **Overview of the flocks diagnosed with colibacillosis, with related metadata and sequence types for each flock**.

Flock ID	Sampling date	Hybrid	Georgraphical location^a^	Sampled birds/flock (n)	ST (n)^b^	ST^c^	FWM (%)^d^
1	05.09.2018	Rowan ranger	South-east	3	2	1112, 155	5.06
3	07.09.2018	Ross 308	South-west	5	2	429, 117	2.29
4	29.10.2018	Ross 308	East	5	3	95, 457, 101	4.50
5	09.11.2018	Ross 308	North-east	5	1	135	2.84
6	13.12.2018	Ross 308	East	5	1	429	3.23
7	17.12.2018	Ross 308	East	5	1	429	4.82
9	03.01.2019	Ross 308	South-west	5	1	429	1.82
10	07.01.2019	Ross 308	East	5	1	429	4.07
11	14.01.2019	Ross 308	North-east	4	1	429	3.33
12	17.01.2019	Ross 308	South-west	4	2	95, 457	4.79
16	14.03.2019	Hubbard	Mid	5	2	93, 95	1.64
22	13.05.2019	Hubbard	Mid	5	3	101, 95, 69	13.7
25	31.05.2019	Ross 308	South-west	5	3	95, 10,836, 457	2.60
27	25.06.2019	Hubbard	Mid	5	2	95, 93	2.36
29	12.08.2019	Sasso	East	5	1	429	3.40
30	21.08.2019	Ross 308	South-west	5	1	371	2.06
32	30.08.2019	Hubbard	Mid	5	2	95, 101	3.07
37	17.02.2020	Ross 308	East	5	4	1611, 1170, 2491, 2040	6.01
38	03.03.2020	Hubbard	Mid	5	1	95	2.06
39	06.03.2020	Ross 308	Mid	5	5	2040, 69, 2753, 95, 10	5.43
42	14.07.2020	Ross 308	South-west	5	1	117	2.98
44	20.08.2020	Ross 308	South-west	5	2	371, 1656	2.31
47	18.09.2020	Ross 308	East	5	4	1684, 69, 10, 5340	6.85
48	21.09.2020	Ross 308	East	5	5	2690, 349, 88, 154, 10	2.92
51	02.10.2020	Hubbard	Mid	5	1	117	12.6
52	15.10.2020	Ross 308	South-west	5	4	191, 1640, 1841, 3006	5.22
53	22.10.2020	Ross 308	South-west	5	4	95, 10, 428, 1146	2.57
54	13.11.2020	Ross 308	East	5	3	1146, 1841, 69	2.25
56	17.03.2021	Ross 308	East	5	1	23	4.70
57	17.03.2021	Ross 308	East	5	1	23	7.74
58	23.03.2021	Ross 308	South-west	5	2	23, 69	2.14
59	23.03.2021	Ross 308	South-west	5	1	23	11.51
62	08.04.2021	Ross 308	South-west	5	1	23	2.00
63	08.04.2021	Ross 308	South-west	5	1	23	3.59
65	13.04.2021	Ross 308	South-west	5	1	23	3.34
69	14.04.2021	Ross 308	South-west	5	1	23	2.72
70	14.04.2021	Ross 308	South-west	5	1	23	2.12
73	19.04.2021	Ross 308	North-east	5	1	23	7.73
74	20.04.2021	Ross 308	East	5	1	23	2.04
78	21.04.2021	Ross 308	East	5	1	23	5.09
80	22.04.2021	Ross 308	North-east	3	2	23	3.80
90	30.04.2021	Ross 308	South-west	5	1	23	3.39
101	06.05.2021	Ross 308	East	5	1	23	4.74
103	11.05.2021	Ross 308	East	5	1	23	4.35
104	14.05.2021	Ross 308	South-west	5	1	23	1.53

^a^The geographical location in Norway for broiler production is divided into five regions; South-west, South-east, East, North-east and Mid Norway.

^b^Number of distinct sequence types (STs) identified in the samples from the representative flock.

^c^Sequence types (STs) present within the flock.

^d^Percent first week mortality (FWM) includes all birds euthanized and succumbed within a flock 7 days post hatching.

### Bacteriological examination

Each sample was streaked onto two blood agar (BA) plates and one heart infusion agar (HIA) and incubated at 37 °C anaerobically, in a CO_2_ chamber and under normal atmospheric pressure, respectively, according to standard procedures for bacterial diagnostics as described in Kravik et al*.* [12]. After 18–24 h of incubation all samples were examined for the presence of *E. coli* and the colony morphology on the three agar plates were described. Bacterial growth was divided into sparse, medium, or rich, and the level of purity of *E. coli* was graded from 1 to 4: Pure growth (1), almost pure growth (2), dominating growth of *E. coli* (3) and mixed culture (4). A grade 2 was given if a few colonies of *Enterococcus* spp. or *Proteus* spp. were present on the agar, together with an otherwise pure culture of *E. coli*. Grade 3 was defined by dominating growth of *E. coli*, but in combination with sparse to medium growth of either *Enterococci* spp., *Proteus* spp. or less growth of a different bacterium. A mixed culture, grade 4, was defined based on growth of a minimum of three different bacteria, where *E. coli* was not the dominating bacterium on the three agar plates (Additional file 1). During bacteriological examination, at least one confirmed *E. coli* isolate from each organ was frozen and stored for future analysis.

### Flock diagnosis

An individual diagnosis of colibacillosis was given based on the presence of pathological lesions typically associated with colibacillosis septicemia in combination with a bacteriological examination graded 1–3. We defined a flock diagnosis if FWM was higher than 1.5% and at least three out of the five sampled birds from the flock were diagnosed with colibacillosis individually.

### Whole genome sequencing

From each flock with a confirmed colibacillosis diagnosis, 3–5 birds per flock were sampled and one isolate from each sampled bird was selected for WGS. Each isolate was preferably isolated from the liver and DNA was extracted as described previously [12]. Genomic DNA samples from 204 isolates were prepared and sequenced at the Norwegian Veterinary Institute (NVI). An additional 15 isolates were included, previously described in Kravik et al. [12]. All 219 isolates were subjected to library preparation: Nextera^™^ DNA Flex library preparation (Illumina), and sequenced on an Illumina MiSeq instrument, resulting in 300 bp paired-end reads. The sequence data analyzed in this study are found publically available in the ENA database with bioprojects PRJEB43441 and PRJEB55163. See Additional file 1 for individual accession numbers.

### In silico analysis

#### Whole genome sequence assembly and typing

The Bifrost pipeline [13] was used for initial quality control and assembly. This pipeline consists of read quality control, trimming, removal of PhiX and assembly. ARIBA [14] version 2.14.6 was used to determine the sequence types (ST) according to the Achtman scheme [7]. Isolates with novel sequence types were uploaded in Enterobase for ST assignment [15]. Serotypes were identified using SerotypeFinder [16] version 2.0.2.

#### Virulence-associated genes

Analysis for detection of VAGs was performed using VirulenceFinder version 2.0.4. The VirulenceFinder database was extended by adding known APEC-associated genes found in the Virulence Factors of Pathogenic Bacteria database (Additional file 2), [2, 17–19]. The complete database consisted of 629 entries of virulence-associated genes and their variants.

#### Core gene analysis and phylogenetic analysis

All isolates that passed QC parameters were included in a phylogenetic analysis based on the core genes. The ALPPACA pipeline [20] version 1.0.0 was used to run genome annotation with Prokka [21] version 1.14.6, followed by pangenome analysis with Panaroo [22] version 1.2.9 to detect and align the core genes among the 219 genomes. Constant sites were removed from the alignment by using Snp-sites [23] version 2.5.1. Snp-dists [24] and version 0.8.2 was used to calculate the pairwise SNP distances from the alignment. Lastly, IQTree [25] version 2.1.4 was used to generate the phylogenetic tree, using Ultrafast bootstrapping [26] with 1000 replicates, and model finder plus [27] for model selection.

Within two of the most frequent STs (ST23 and ST429) separate phylogenetic analyses with ALPPACA were performed. ParSNP [28] version 1.6.1 was used to generate a core genome alignment, followed by detection of recombinant regions with Gubbins version 3.2.0 using RaxML as the treebuilder and the GTRGAMMA model. Maskrc-svg [29] version 0.5 was subsequently used to mask recombinant areas from the alignment. Constant sites were removed by using Snp-sites, followed by pairwise SNP distance calculation with snp-dists and phylogenetic inference with IQTree, similar to above. All phylogenetic trees were visualized in R [30] version 4.0.2, using the ggtree package version 3.0.4 [31].

## Results

### Sampling and colibacillosis confirmation

From September 2018 to June 2021, 45 broiler and four broiler breeder rearing flocks were sampled, resulting in a total of 49 flocks with FWM ranging from 1.53% to 12.6%. The flocks were of different hybrids, all less than 14 days of age, from various regions in Norway and originated from three different hatcheries. Four broiler flocks were not diagnosed with colibacillosis, and thereby excluded from further analysis. Altogether, 45 flocks with high FWM were given a colibacillosis diagnosis and further included in the analysis (Table 1).

### Whole genome sequencing and in silico analysis

#### Quality control

Altogether 219 confirmed *E. coli* isolates were sequenced. One *E. coli* was selected from each bird, and 3–5 birds were sampled from 45 flocks (Additional file 1). Initial quality control of the genome sequences, based on multiQC and Quast report, showed that the GC content of the isolates were between 50.51 and 50.57%, the number of contigs were 43–74 and the total length of the complete genome after assembly was 4.86–4.96 Mbp (See individual quality scores for all isolates in Additional file 1).

#### MLST and serotyping

The 219 APEC isolates from 45 flocks were sequenced and characterized by ST and in silico serotype. Twenty-six of these flocks exhibited the same ST in all isolates from within a flock, while 35 flocks were identified with up to two STs and therefore identical ST in a minimum of three birds from within the same flock (Table 1 and Additional file 1). Ten of the 45 flocks were identified having three or more STs within the flock (Table 1).

Altogether 32 different STs were identified, of these, 15 were only identified once. The most common STs identified in this study were ST23, ST429, ST95, ST117, ST371, ST69 and ST101 in descending order (Table 2).

**Table 2:** **Description of the ten most common sequence types identified**.

ST	Isolates (n)	Flocks (n) where ST is identified	Flocks (n) where ST is identified in > 3 isolates	Serotypes
23	81	17	17	O78:H4
429	33	7	7	O2/O50:H1
95	24	10	3	O1:H7, O2/O50:H5
117	11	3	2	O161:H4, O24:H4, O78:H4
371	9	2	2	O45:H19
69	8	5	0	O15:H6, O17/O44/O7:H18, O23:H6
101	7	3	1	O103:H21, O88:H8
135	5	1	1	O2/O50:H1
10	4	4	0	O49:H12, O71:H40, O99:H33
457	4	3	0	O11:H25
93	4	2	1	O5:H10

In silico serotyping revealed a total of 38 distinct serotypes. Some serotype profiles were detected within a single ST, whereas other serotype profiles were detected across several STs. Of the ten most common STs, five STs were identified with several serotype profiles. The most common serotypes were O1:H7, O2/O50:H1, O2/O50:H5, O45:H19 and O78:H4 (Table 2).

#### Phylogenetic analysis

**Figure 1:**
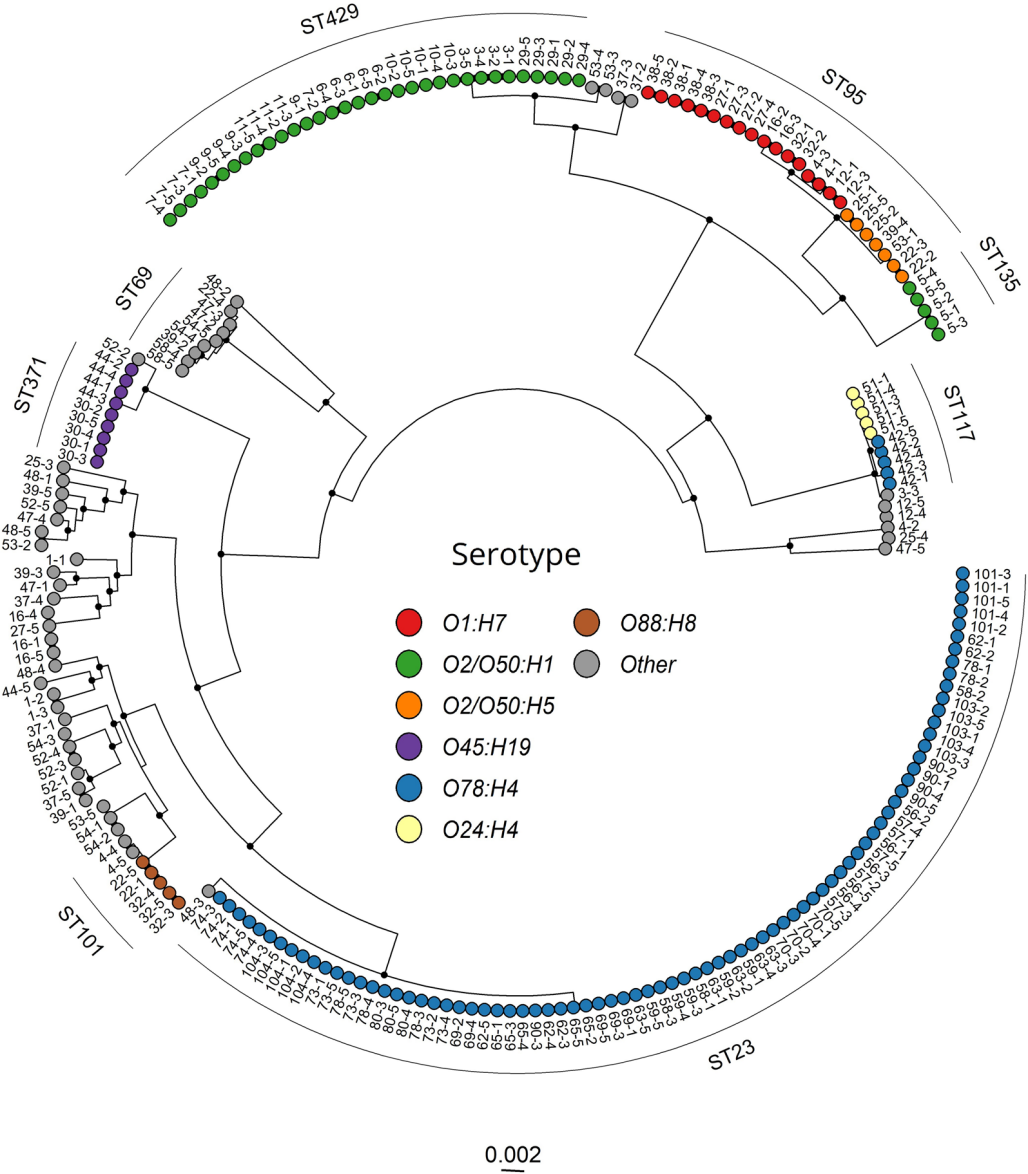
**Maximum likelihood core-gene SNP tree of all isolates included in the study.** Bootstrap values above or equal to 95 are denoted as black nodes. The core gene tree visualizes the genetic relations between the most common sequence types (STs) identified and what serotype profiles are linked to these. Colors on the tips and clade labels represent serotypes and STs, respectively, represented by more than five isolates. Tip labels represent flock and bird.

To investigate the relationship across STs and between STs and serotypes, a core gene analysis of all 219 isolates was performed (Figure 1). Among the 219 isolates, the pangenome analysis detected 14,332 unique genes. Out of these, 3303 were defined as core genes as they were present in at least 95% of the genomes. Model finder plus identified GTR + F + I + G4 as the best-fitting model. The phylogenetic tree revealed that the isolates clustered according to their STs and it showed that isolates with the same serotype profile might be genetically distant. Isolates of serotype O78:H4 were identified as both ST23 and ST117, and serotype O2/O50:H1 was identified as both ST429 and ST135. Further, ST95 and ST117 also contained several serotype profiles: O1:H7 and O2/O50:H5 and O24:H4, O78:H4 and O161:H4, respectively.

**Figure 2:**
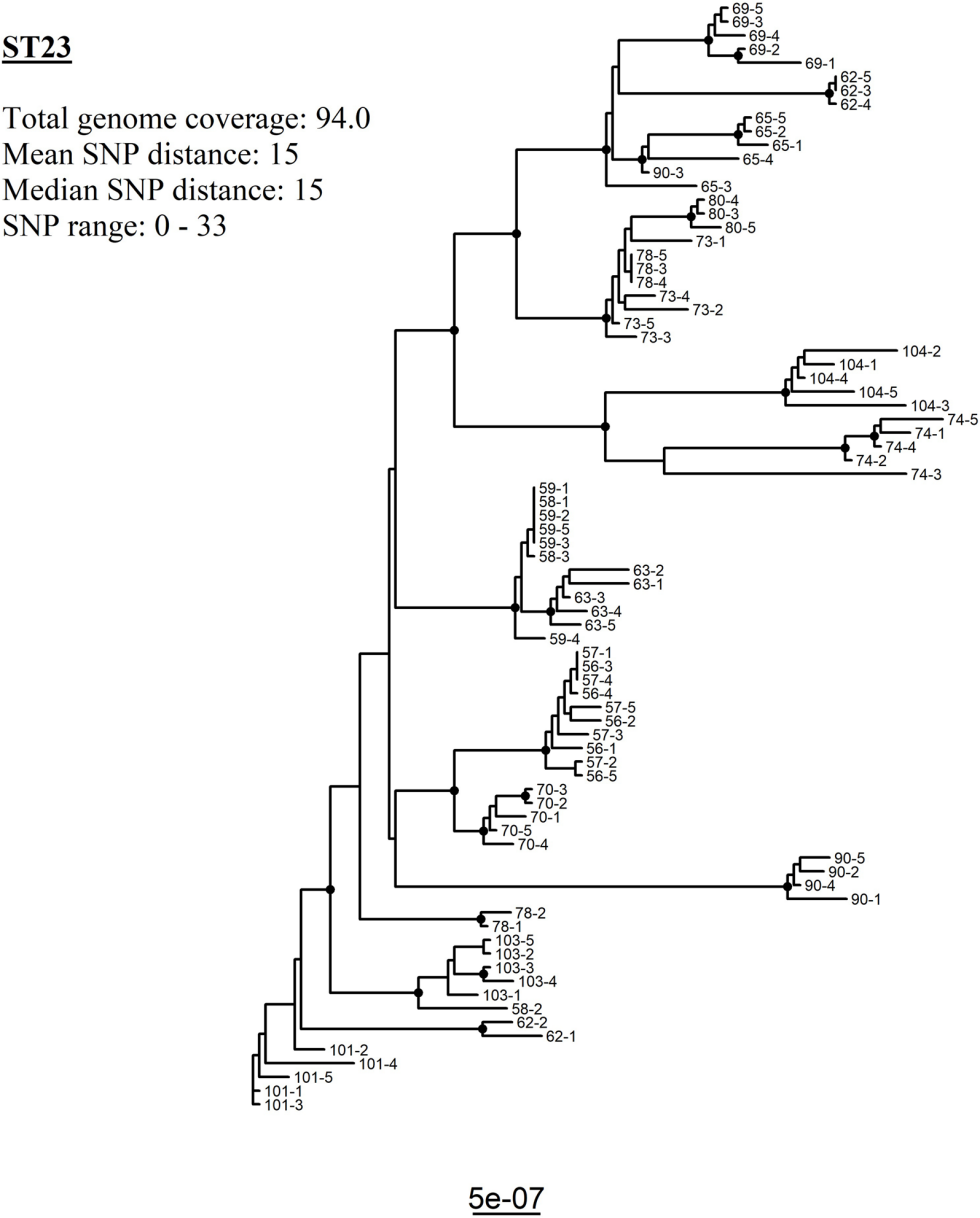
**Maximum likelihood core genome tree visualizing the genetic relations of all isolates identified as ST23 from 17 flocks (n = 81 isolates).** Bootstrap values above or equal to 95 are denoted as black nodes. Tip labels represent flock and bird.

**Figure 3:**
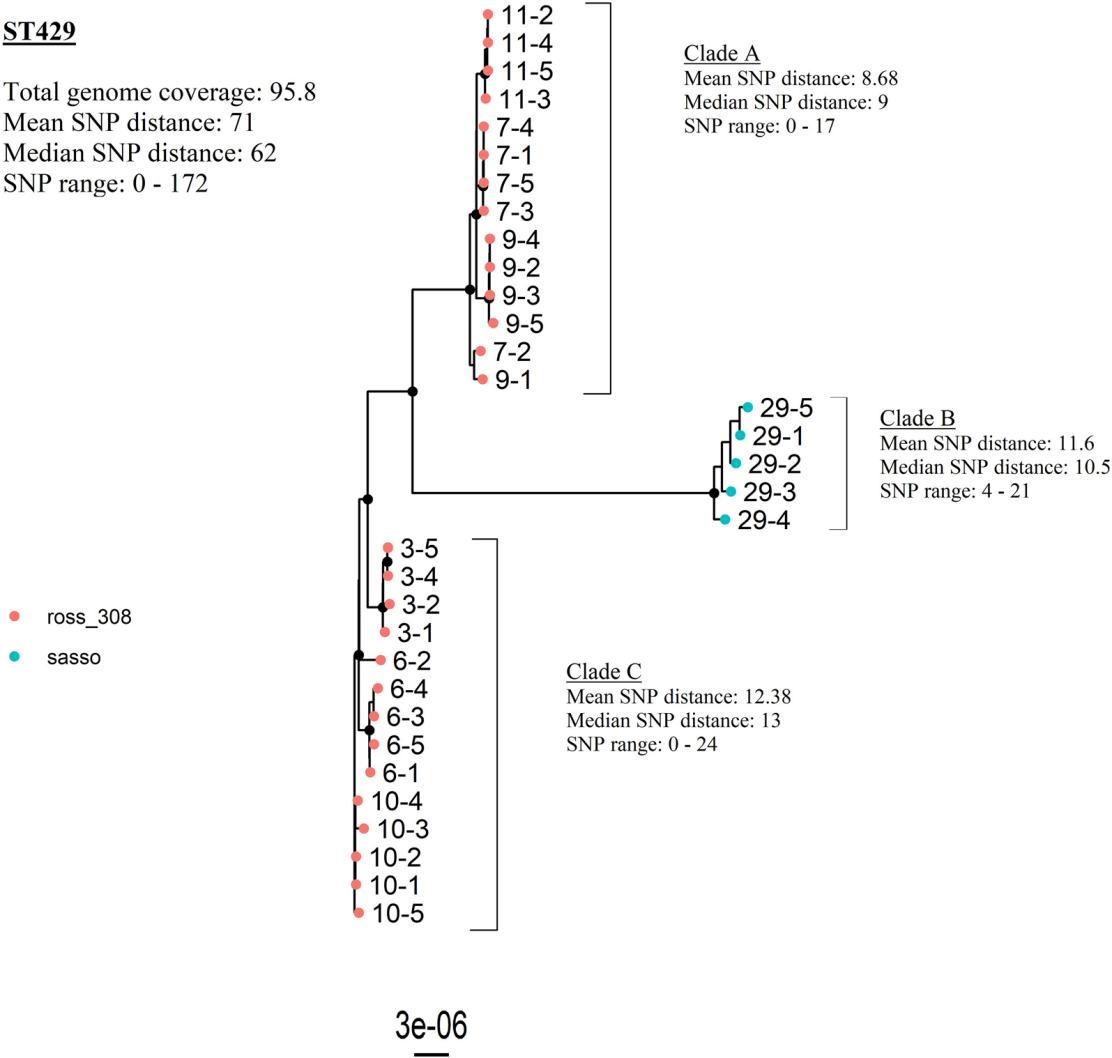
**Maximum likelihood core genome tree of all isolates identified as ST429 from seven flocks (n = 33 isolates).** Bootstrap values above or equal to 95 are denoted as black nodes. Tip labels represent flock and bird. Colors on the tips represent hybrid. Clade A and C consists of isolates from three flocks each, all of hybrid Ross 308. Clade B represents a single Sasso flock.

The two largest clusters in the core gene tree were represented by ST23 and ST429, respectively. Both STs had one distinct serotype profile and all flocks, except one flock with ST429, clustered according to hybrid, sampling dates and ST (Figure 1, Table 1). These two STs were therefore suspected to represent two outbreaks of colibacilllosis and separate phylogenetic analyses were therefore carried out for each of them (Figures. 2 and 3).

A total of 81 isolates from 17 flocks were confirmed to be of ST23. All ST23 isolates were collected from broiler flocks of the hybrid Ross 308 and sampled from March 2021 until the end of May 2021 (Table 1). The phylogenetic analysis of ST23 had an average genome coverage of 94.0%, a SNP range of 0–33 and a mean and median SNP distance of 15. Individual isolates of ST23 clustered, for the most part, according to individual flocks, but there were also isolates which clustered with isolates from different flocks (Figure 2 and Table 3). Within a flock, all but two flocks had mean SNP distances below 10. The SNP range varied from 0 to 1 (smallest range) to 0–33 (highest range) within a flock (Table 3).

**Table 3:** **Overview of the calculated SNP distances between isolates of same sequence type (ST23 and ST429) within a flock**.

Flock ID	ST	Mean SNP distance	Median SNP distance	SNP range	Isolates/flock (n)
56	23	1.2	1	0–2	5
57	23	1.2	1	0–2	5
58	23	8.7	13	0–13	3
59	23	0.4	0	0–1	5
62	23	19.8	32	0–33	5
63	23	2.4	2.5	1–4	5
65	23	7.4	7	1–13	5
69	23	6.6	7	1–11	5
70	23	1.4	1.5	0–2	5
73	23	2.4	2.5	1–4	5
74	23	7.4	3	1–17	5
78	23	7.6	12	0–13	5
80	23	0.6	1	0–1	3
90	23	13.1	3	1–31	5
101	23	2.2	2.5	0–4	5
103	23	2	2	0–3	5
104	23	3	4	1–7	5
3	429	3.3	4	0–5	4
6	429	8.8	5	2–19	5
7	429	4.4	0	0–11	5
9	429	6.8	2	0–17	5
10	429	2.8	2.5	0–6	5
11	429	0.7	1	0–1	4
29	429	11.6	10.5	4–21	5

ST429 was identified in 33 isolates from seven flocks; six flocks of the hybrid Ross 308 sampled between September 2018 to January 2019, and one flock of the hybrid Sasso sampled in August 2019. The phylogenetic analysis revealed an average genome coverage of 95.8% and the SNP range was 0–172 with a median SNP distance of 62. The tree diagram shows that ST429 isolates separate into three dominating clades: ST429-A, ST429-B and ST429-C (Figure 3). Isolates from the only Sasso flock clustered together in clade ST429-B. Isolates in clade ST429-A and ST429-C originated from Ross 308 flocks from different geographical locations, hatcheries and parent flocks, however, all Ross 308 broiler rearing flocks are distributed from one main hatchery (Figure 3). Within the individual flocks identified with ST429, the mean SNP distances were in all cases < 10 SNPs, except from the Sasso flock, and the SNP range varied from 0 to 1 to 4–21 (Table 3).

The phylogenetic analysis of ST429 was re-run, including only flocks of hybrid Ross 308. The results from this analysis showed an average genome coverage of 95.7% and the SNP range was 0–76 with a median SNP distance of 59 (Additional file 3).

#### Virulence-associated genes

**Figure 4:**
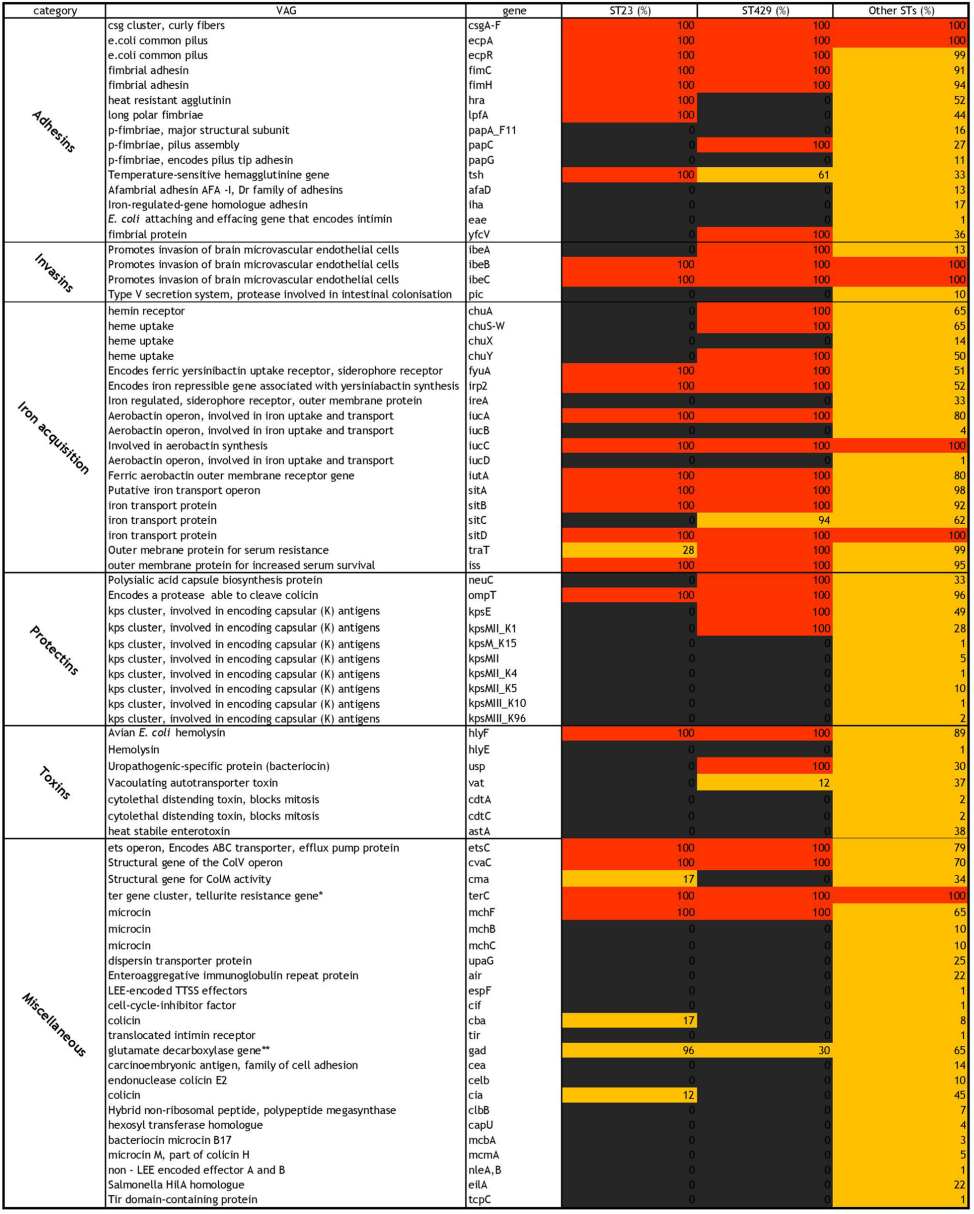
**Frequency of virulence-associated genes (VAGs) present in three groups of isolates: ST23 (n = 81), ST429 (n = 33) and a third group comprising the remaining isolates (n = 105).** The colors represent the frequency from which each gene is present: red = 100%, black = 0% and yellow = 1–99%. The VAGs are categorized and described as previously presented by Nolan et al. [1] and Kathayat et al*.* [18].

From a database containing 629 entries of VAGs, 112 VAG-variants were identified in at least one of the APEC isolates from the present study (Additional file 1). The frequency of VAGs was described separately for isolates of ST23 and ST429 and a third group that included all other isolates (Figure 4).

A total of 25 VAGs were identified in all isolates of ST23, while five VAGs were identified in only some of the ST23 isolates. These include *tra*T, *cba*, *cia* and *cma* that were identified in only 28%, 17%, 12% and 17% of these isolates, respectively. The *gad* gene was identified in 94% of all the ST23 isolates (Figure 4). Thirty three of the VAGs were present in all isolates of ST429, while four genes were found in some isolates. The *tsh*, *sitC*, *vat* and *gad* genes were identified in 61%, 94%, 12% and 30% of the ST429 isolates, respectively (Figure 4).

In the third group containing all other STs, there was higher diversity in the presence of VAGs. Similar to ST23 and ST429, all of the isolates in the third group carried the genes *csg*A-F, *ecp*A, *ibe*B, *ibe*C, *iuc*C, *sit*D and *ter*C. Several other VAGs were present in more than 90% of all the isolates in the third group, but there were also VAGs that were absent or only present in a few isolates (Additional file 1 and Figure 4). The siderophore receptor-genes *fyu*A and *irp*2 were present in all ST23 and ST429 isolates, but only present in 51% and 52% of the other isolates, respectively.

## Discussion

There is limited knowledge about APEC in the Norwegian broiler production, and after the peak of outbreaks of colibacillosis on farms in the Nordic countries in 2014, we saw the need for a systematic study of the epidemiology of APEC in the Norwegian broiler production. To identify APEC types within the Norwegian broiler production, we performed systematic sampling of flocks with high FWM and sequenced the genomes of individual isolates from these flocks to study their STs, serotype profile, content of VAGs and their evolutionary relationship.

The selection of isolates was carried out according to the results from a pilot project in 2019. The study concluded (1) low diversity of APEC within individual diseased birds, and (2) the need of sampling a minimum of three birds per flock to identify the diversity of APEC within a flock and determine the main disease-causing APEC at flock level [12].

Out of the 32 distinct STs identified in the present study, almost 50% were only identified once. A similar trend has been observed in previous studies [32–34]. The high number of STs only represented by one or two isolates influences the high diversity of STs reported. Whether these single ST strains are true avian pathogens and important for the colibacillosis etiology, or sporadic findings without importance for pathogenicity, is currently not known. These reports, however, highlight the need for sampling multiple animals and WGS of several isolates from a flock to identify the main disease-causing APEC within a flock.

Altogether, 35 of 45 diseased flocks were identified with a dominating APEC type, and ST23, ST117, ST371 and ST429 were more often found to cause disease in a flock alone than other STs. The latter indicates that these STs are possibly more pathogenic in poultry, compared to the STs more commonly identified in mixed infections with more than one ST within a flock [35]. Altogether, ten flocks exhibited a combination of several STs and the APEC types most commonly identified in combination with other STs were ST10, ST69, ST95 and ST101.

Notably, both ST69 and ST95 are frequently isolated in human infections [5, 36]. However, due to their presence in a majority of mixed infections, it is likely to assume that they are rather opportunistic than highly pathogenic in poultry. This is supported by Kromann et al. where ST95 was identified with the highest prevalence from healthy poultry flocks sampled in a non-outbreak situation [37].

Caution should be taken, however, in the discussion of ST95 as this ST is identified with several serotype profiles, and variants of the same ST but with different virulence properties, exists [5, 36, 37]. Our study shows further examples of STs with several serotype profiles, such as ST101 where O88:H8 was identified from three flocks and O103:H21 from another flock and ST117, which exhibits three serotypes O24:H4, O78:H4 and O161:H4 from three different flocks. Serotype O78:H4 is the same serotype as found in ST23. Generally, typing of APEC should be evaluated with caution, as shown in the core gene analysis where ST117 and ST23 exhibit large genetic distances between these STs, even though they share one of the same serotype profiles. Without WGS data, the two distinct peaks of ST117 in 2014 [11] and ST23 in 2021, with possible distinct origins, would be considered the same APEC if only serotyping was performed, but two distinct APEC types if MLST was performed. Sequence types identified with several serotype profiles, on the other hand, suggests that a combination of two typing methods provides better differentiation between APEC isolates (for quick diagnosis in outbreak situations) [35].

Even though APEC is considered a diverse pathogen, there is less variation amongst the most frequent STs reported [12, 33, 35, 38–40]. The high prevalence of certain STs, however, may be due to small outbreaks of colibacillosis from a single source and within a limited period, possibly from higher up in the production pyramid [41].

Phylogenetic analysis is today considered the gold standard to evaluate relatedness between isolates from a possible single source. Accordingly, this study presents the mean SNP-distance within, and between flocks identified with the same ST. The results are valuable for the evaluation and understanding of future outbreaks of colibacillosis across farms. To our knowledge, there is no consensus for defining the acceptable number of SNPs in APEC outbreaks [9, 10, 12]. Factors such as the mutation rate, the number of individuals the pathogen might encounter, pathogen pressure and the duration of source contamination could influence the number of SNPs emerging during an outbreak [9]. The consideration of SNP distances between isolates from an outbreak, should therefore, include the pyramidal structure of the poultry production and the number of individuals and generations the pathogen will encounter in a potential vertical transmission line, before causing disease in a broiler chick. Further, SNP distances alone should not be assessed without the knowledge of the proportion of the genome that has been used in the analysis. Therefore, pathogen- and population metadata, as well as genome coverage, plays as much a role in the understanding if an isolate belongs to an outbreak as the SNP distance alone [9, 10, 12].

Altogether, 112 VAGs were identified at least once in our set of APEC isolates. This correlates well with recent reports by Apostolakos et al*.*, who identified 113 VAGs in their study [32]. However, within each of the two main STs (ST23 and ST429) identified in the present study, there was, with few exceptions, less diversity. This is expected as the isolates within these STs were considered part of the same outbreaks, and therefore likely to be clonal. The genes varying within ST23 were *traT*, *cba*, *cia* and *cma*, and within ST429 *vat* and *gad.* Most of these genes are known to be plasmid-encoded and might therefore vary more frequently between isolates within an outbreak than chromosomally encoded genes. Further, *fyuA* and *irp2* were represented in all outbreak isolates, but only in approximately half of the non-outbreak isolates. Both these VAGs belong to the Yersiniabactin operon which is responsible for iron acquisition and of high importance in the pathogenesis of avian colibacillosis [1].

A few VAGs were, on the other hand, identified in all isolates in this study and could therefore be considered important for the virulence of APEC. However, without a true, non-pathogenic control group for comparison, no valid conclusions may be drawn based on these data as these VAGs might be identified in all avian *E. coli* isolates regardless of pathogenicity. A recent study from Johnson et al. [35] suggested two conserved VAGs associated with APEC plasmid, *hlyF* and *ompT* as potential markers for increased virulence potential in combination with other genetic features. In the present study, *hlyF* and *ompT* were identified in all ST23 and ST429 isolates and in 89 and 96% of isolates of other STs, respectively.

In this study, the virulence of an APEC strain was evaluated based on the prevalence of the identified ST and its’ ability to cause high FWM within a flock and across farms alone or in combination. Further, we have given insight into the relatedness of outbreak strains, and presented the most prevalent VAGs associated with the two outbreak strains ST23 and ST429 in our study. However, APEC strains emerge from multiple *E. coli* lineages, and for the future it would be interesting to follow single ST outbreaks to identify if the same ST reappear as more prone to cause outbreaks across farms or if other STs, which in this study appear of lesser importance, could be the cause of future outbreaks. It would be interesting to better identify the transmission routes of the pathogen in outbreak situations between farms. A common database for the control and prevention of APEC outbreaks has been suggested [42]. For the future, the authors supports an initiative for such a database, including well-defined metadata as well as comparable sampling and diagnostic methods. Such a database could aid in the identification of pathogen transmission routes through the broiler poultry pyramid. Further, the comparison of VAGs from systematically sampled outbreak strains to a proper control group would be of interest to further unravel the virulence potential of individual APEC. The importance of defining what an APEC control is, should however, be further discussed as commensal *E. coli* might have the potential to become APEC and the pathophysiology behind is still not well defined [38].

In conclusion, this study shows the presence and distribution of APEC types identified from local outbreaks of colibacillosis-septicemia across Norway during 2018–2021. Further, it identifies how peaks of high FWM due to colibacillosis may be caused by a single, distinct ST. Phylogenetic analysis gives insight into the relatedness between isolates belonging to the same ST, but also across STs and serotypes, identifying the need for combining typing methods to better discriminate between APEC types. This study also highlights the value of using WGS as a diagnostic tool for surveillance as well as in the identification of future outbreaks of colibacillosis.

## Supplementary Information

**Additional file 1. Metadata linked to each isolate sequenced in this study including flock and sampling data, bacteriological grading, sequencing quality measures, virulence-associated genes identified and accession numbers to ENA.** ^1^The sample ID explains from what flock (first number) and bird (last number) the isolate was sampled. ^2^The bacterial culture was graded according to purity of growth on three agar plates incubated over night in either normal oxygen pressure, CO2 chamber or anaerobically: grade 1 = pure growth, grade 2 = few colonies of *Enterococcus* spp. or *Proteus* spp. were present on the agar, together with an otherwise pure culture of *E. coli.* Grade 3 = dominating growth of *E. coli* with sparse to medium growth of either *Enterococci* spp, *Proteus* spp. or less growth of a different bacteria, grade 4 = mixed culture: minimum of three different bacteria, where *E. coli* was not the dominating bacteria. ^3^The presence of pathological lesions associated with colibacillosis (colisepticaemia) within an individual bird during necropsy, 1 if ≥2 lesions present, 0 if <2 lesions present. ^4^Each individual bird received a colibacillosis diagnosis (1) if the bird had lesions of colisepticaemia and the bacteriological examination from the individual sample was graded 1-3.

**Additional file 2. List of genes included in the extended database of genes uploaded to VirulenceFinder for the identification of virulence-associated genes in this study.** Included in the table is the name of gene, short description of gene and from what database the gene was identified (VirulenceFinder or VFDB).

**Additional file 3. Maximum likelihood core genome tree visualizing the genetic relations of all isolates identified as ST429 from all Ross 308 flocks (n = 28), excluding isolates from one Sasso flock.** Bootstrap values above or equal to 95 are denoted as black nodes. Tip labels represent flock and bird. Clade A and C consists of isolates from three flocks each, all of hybrid Ross 308.
