# Supplementary figures and images for: A molecular epidemiological study on Escherichia coli in young chicks with colibacillosis identified two possible outbreaks across farms
Source: Vet Res. 2023 Feb 6;54:10. doi: 10.1186/s13567-023-01140-6 (PMC9901153; doi:10.1186/s13567-023-01140-6)

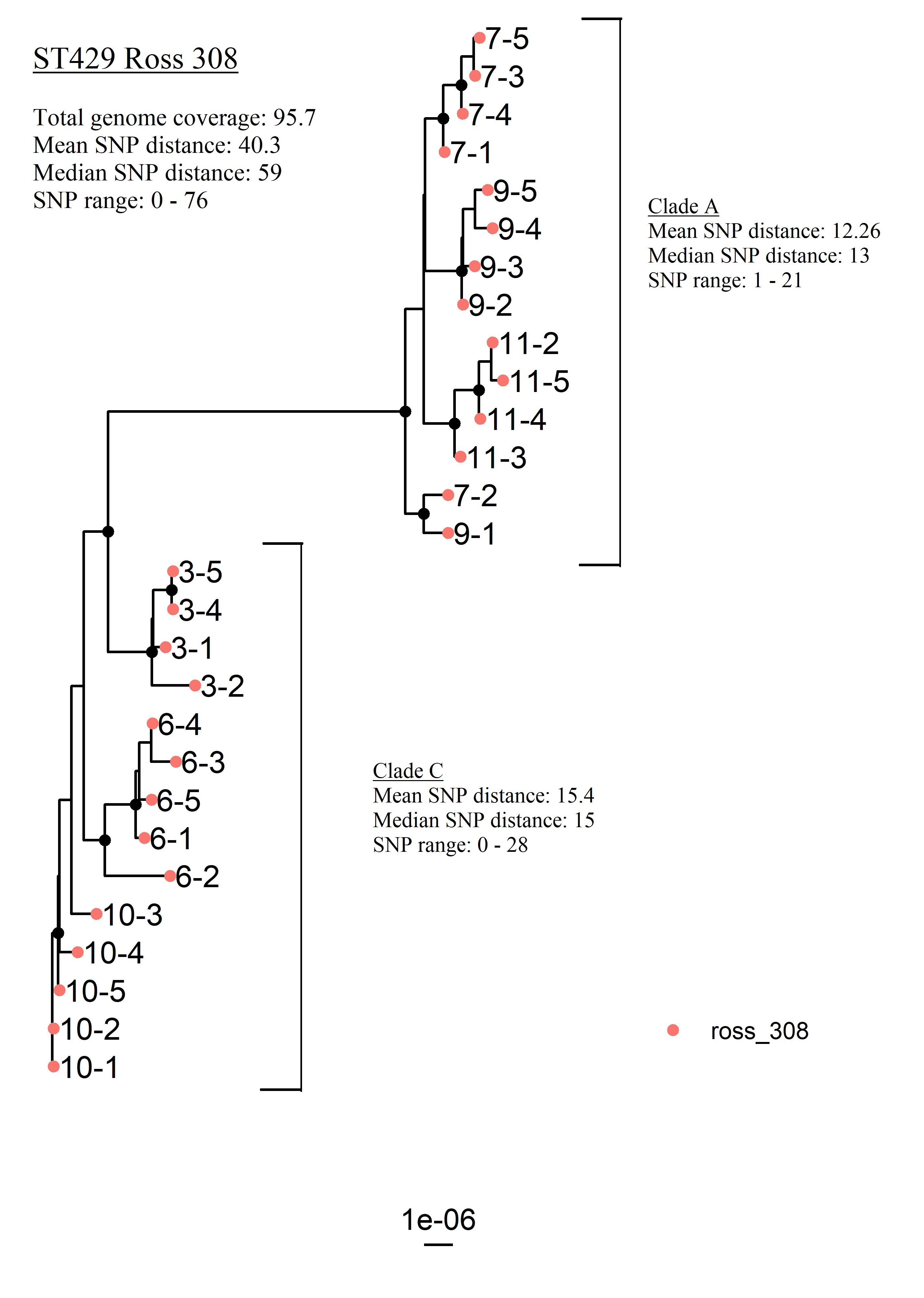

Supplement: Supplementary file 3 — Additional file 3. Maximum likelihood core genome tree visualizing the genetic relations of all isolates identified as ST429 from all Ross 308 flocks (n = 28), excluding isolates from one Sasso flock. Bootstrap values above or equal to 95 are denoted as black nodes. Tip labels represent flock and bird. Clade A and C consists of isolates from three flocks each, all of hybrid Ross 308. [file 13567_2023_1140_MOESM3_ESM.jpg]
